# Household cost of malaria overdiagnosis in rural Mozambique

**DOI:** 10.1186/1475-2875-7-33

**Published:** 2008-02-18

**Authors:** Jen CC Hume, Guy Barnish, Tara Mangal, Luiz Armázio, Elizabeth Streat, Imelda Bates

**Affiliations:** 1 Disease Control Strategy Group, Liverpool School of Tropical Medicine, Liverpool, UK; 2 Laboratory of Malaria and Vector Research, NIH/NIAID, 12735 Twinbrook Parkway, Rockville, MD 20892, USA; 3 District Director of Health, Mocuba, Zambézia Province, Mozambique; 4 Maputo Province Directorate of Health, LSDI Malaria Program, Matola City, Maputo Province, Mozambique

## Abstract

**Background:**

It is estimated that over 70% of patients with suspected malaria in sub-Saharan Africa, diagnose and manage their illness at home without referral to a formal health clinic. Of those patients who do attend a formal health clinic, malaria overdiagnosis rates are estimated to range between 30–70%.

**Methods:**

This paper details an observational cohort study documenting the number and cost of repeat consultations as a result of malaria overdiagnosis at two health care providers in a rural district of Mozambique. 535 adults and children with a clinical diagnosis of malaria were enrolled and followed over a 21 day period to assess treatment regimen, symptoms, number and cost of repeat visits to health providers in patients misdiagnosed with malaria compared to those with confirmed malaria (determined by positive bloodfilm reading).

**Results:**

Diagnosis based solely on clinical symptoms overdiagnosed 23% of children (<16y) and 31% of adults with malaria. Symptoms persisted (p = 0.023) and new ones developed (p < 0.001) in more adults than children in the three weeks following initial presentation. Adults overdiagnosed with malaria had more repeat visits (67% v 46%, p = 0.01–0.06) compared to those with true malaria. There was no difference in costs between patients correctly or incorrectly diagnosed with malaria. Median costs over three weeks were $0.28 for those who had one visit and $0.76 for ≥ 3 visits and were proportionally highest among the poorest (p < 0.001)

**Conclusion:**

Overdiagnosis of malaria results in a greater number of healthcare visits and associated cost for adult patients. Additionally, it is clear that the poorest individuals pay significantly more proportionally for their healthcare making it imperative that the treatment they receive is correct in order to prevent wastage of limited economic resources. Thus, investment in accurate malaria diagnosis and appropriate management at primary level is critical for improving health outcomes and reducing poverty.

## Background

In sub-Saharan Africa over 70% of patients with suspected malaria diagnose and manage their illness at home with traditional remedies or drugs bought from local shops [1]. Attendance at a health clinic usually occurs only after home treatment has failed. Primary level health facilities lack tools for detection of malaria parasitaemia so a diagnosis of malaria is usually based on clinical symptoms such as fever. Despite the development of algorithms to improve the specificity of clinical malaria diagnosis, many infectious diseases mimic malaria illness so this approach leads to over diagnosis rates of 30–70% depending on malaria transmission patterns [1,2]. Overdiagnosis of malaria leads to unnecessary antimalarial drug use, increased drug resistance, and delays in achieving the correct diagnosis: all of which prompt return visits to health facilities.

Malaria misdiagnosis can either result in over estimating or under estimating the burden of disease and in both instances the burden of malaria misdiagnosis is likely to fall most heavily on the poor and vulnerable because they have less resilience to cope with the effects of prolonged ill health [1]. There is almost no evidence available about the economic impact of malaria misdiagnosis on households although there is information about the costs of malaria episodes. Very low income households in Malawi spent 32% of their annual household income on direct and indirect costs for malaria illness compared to 4.2% in more wealthy households [3]. In Sri Lanka, some families spent more than 10% of their annual household income per malaria episode [4] and children lost 10% of school days [5]. Indirect costs, such as transport and days off work for carers represented 79% of total costs of each malaria episode in Ghana [6].

This study documented the number and cost of repeat consultations with health providers in rural Mozambique specifically as a result of malaria overdiagnosis. Such information is important because many countries in sub-Saharan Africa are introducing, or have recently introduced, new malaria drug policies based on combination therapies. These combinations are more expensive than previous first-line drugs such as chloroquine and sulphadoxine-pyrimethamine (SP) and using them to treat illnesses that are not truly malaria will be a significant waste of resources as well as having a detrimental impact on health outcomes and drug resistance. Recommended malaria treatment in Mozambique at the time of this study was amodiaquine and SP (AQ/SP).

High rates of overdiagnosis of malaria are well recognised but have been tolerated because first-line antimalarial drugs, such as chloroquine, were cheap. The introduction of more expensive malaria treatments prompts the need for better knowledge about the consequences of malaria misdiagnosis to improve targeting of malaria drugs to those who truly have malaria illness and to make sure that the poorest benefit from these highly effective medicines [7].

## Methods

### Study area

The study was conducted in Mocuba in central Mozambique, a rural town 80 km from the Zambézia Provincial capital of Quelimane with a population of 66,000. Malaria is Mozambique's leading cause of morbidity and mortality, with an estimated 44–67,000 deaths per annum across all age groups [8]. The entire country is endemic for malaria with perennial transmission in the Zambézia Province peaking in January and April/May. Over 90% of reported malaria cases are due to *Plasmodium falciparum*. Mocuba town has a district hospital and two health centres – one government-owned and one private – which only deliver out-patient care. The study was conducted at both health centres in order to maximise patient enrolment.

### Study design

This was an observational study to document the impact on patients of malaria overdiagnosis that occurs during routine malaria care. Patients who attended the clinics were seen by a triage nurse and those with suspected malaria were diagnosed clinically and treated according to local protocols; all attempts were made not to interfere with local practice, allowing clinic staff to diagnose and treat patients as was customary. A full patient history and temperature reading was taken from all patients. Only first-line treatment with anti-malarial drugs is administered at this level; severe cases are transferred to the district hospital. Patients that had recently been diagnosed with malaria and were returning to the clinic for follow-up tests were excluded from the study. All patients who had a clinical diagnosis of malaria, and who consented to take part in the study, provided a finger prick blood sample for malaria slides for microscopy. This was the only change we made to the routine care of patients at the clinic and was necessary to enable us to determine retrospectively which patients truly had malaria; customarily, slides were only taken in less than 10% of cases. Results from any slides requested as part of routine clinical care were processed as normal and results given to triage nurse on the same day. However the results of the blood slides did not appear to alter the treatment regime as patients with negative results were still prescribed anti-malarials based on the clinical diagnosis. The proportion of clinically diagnosed malaria patients who had malaria parasites was determined by microscopy of Giemsa-stained thick blood films by two independent researchers. Discrepant results were resolved by an independent third microscopist.

### Selection of study subjects

This study compared the household costs associated with repeated health-related consultations between patients correctly and incorrectly diagnosed with malaria in the two health centres. Individuals attending either health clinic in May-June 2004 with malaria clinically diagnosed by the triage nurse, and resident within 10 km of the health clinic (to facilitate patient follow up) were invited to enrol in the study. Clinical diagnosis was based on the presence of symptoms and fever at the time of enrolment, according to the local protocol. Both adults and children were enrolled, with children defined as those under 16 years of age and dependent on parental support.

### Ethical approval

Permission for inclusion in the study was sought from all adult participants, and from carers of child patients. The Liverpool School of Tropical Medicine Research Ethics Committee, the Director of Mozambique's National Malaria Control Programme and the Zambézia Provincial Medical Director granted ethical approval for the study.

### Sample size calculation

After discussions with local health workers, a mean difference of 0.15 health consultations per patient per month was considered to have important implications for local public health. This figure was based on two malaria-related health consultations per patient per annum. For the study to have a 90% power (1-probability of a type II error) to detect a difference of this size at a significant level of 5% (probability of a type I error) the total number of study patients required was 466.

### Data collection and variables studied

Three questionnaires were used for data collection. The first at day 0 when the patient first presented at the clinic and then on days 7 and 21 when subjects were visited at home. For children, questionnaires were completed by the accompanying carer. Three weeks was chosen as the period for follow up because this was sufficient time for any self-limiting infection to have resolved and for any underlying non-malarial cause of fever to have become manifest and to have promoted repeat clinic visits. The baseline questionnaire collected data on: age, sex, education level (adults only), occupation (adults only), symptoms, transport and costs, missed wages (either to the participant directly or to any family member accompanying a sick individual), treatments and cost, and monthly household outgoings. Monthly expenditure rather than monthly income was collected as a pilot study indicated that many individuals were unwilling to provide reliable income data and that expenditure information would be more accurate. Although chosen to be more accurate, household expenditure remains a very difficult entity to measure [9] and data was collected to provide an overview rather than to conduct detailed analyses. The second and third questionnaires collected additional data on: persistence of initial symptoms, any additional symptoms and number and cost of all health-related interactions (HIs) either with the initial clinic or with other formal and informal healthcare providers (e.g. dispensary, shop, traditional healer, hospital, other clinic, from relative). Financial information was obtained in Mozambican Meticais (Mt) and converted to US Dollars (US$) using the exchange rate at the time (23,000 Mt equivalent to US$1.00).

### Statistical analysis

The number of HIs by correctly and incorrectly diagnosed individuals over the 21-day study period were compared by fitting them to a Poisson model using the maximum likelihood method and adjusting for overdispersion. P values that were close to the significance threshold are presented as a range with the lower value representing no overdispersion and the higher value representing adjustment for maximum overdispersion. Costs were compared by Mann-Whitney Tests. Analyses were conducted in SigmaStat version 3.1 and STATA version 8.

## Results

535 patients were enrolled in the study; 170 from the private clinic and 365 from the government clinic. After the 21 day follow up and exclusion of those who did not meet the inclusion criteria (specifically those individuals who lived further than 10 km from the health centre than reported) were correlated with the readable slide information, data was available for 312 patients. Children constituted the majority of study participants (65%; 204/312) with most individuals enrolled at the government clinic (69%; 216/312) (Table 1). Because of referrals from ante-natal clinics 71% of adults enrolled at the private clinic and 66% at the government clinic were women. There was no difference between the clinics in the age of adult participants (p = 0.095, Mann-Whitney U test) but the average age of children was higher at the private clinic (p = 0.027, Mann-Whitney U test). 94% of adult participants were subsistence farmers.

**Table 1:** Characteristics of participants with clinically diagnosed malaria.

**Description**		**Private**		**Government**		***P *value**
			
		**Child**	**Adult**	**Child**	**Adult**	
**Sex**	Male (%)	25 (41)	10 (29)	69 (48	25 (34	*P*^1 ^= 0.711
	Female (%)	36 (59)	25 (71)	74 (52)	48 (66)	*P*^2 ^= 0.424
	TOTAL	61	35	143	73	

**Age (years)**	Mean	3.03	33.57	2.6	30.15	*P*^1 ^= 0.095
	Range	0.5–13	18–60	0.2–14	16–61	*P*^2 ^= 0.027

**Occupation**	Unemployed (%)		3 (9)			
	Subsistence farmer (%)		11 (31)		4 (5)	
	Small trader (%)		4 (11)		21 (29)	
	State employee (%)		5 (14)		5 (7)	
	Professional (%)	N/A	3 (9)	N/A	10 (14)	
	Student (%)		4 (11)		2 (3)	
	Housewife (%)		4 (11)		10 (14)	
	Retired (%)		0		10 (14)	
	Other (%)		0		1 (1) 5(7)	
	Declined answer (%)		1 (3)		5(7)	

**Malaria microscopy**	Positive (%)	83 (78)	43 (68)	197 (76)	74 (70)	*P*^1 ^= 0.968
	Negative (5)	24 (22)	20 (32)	62 (24)	32 (30)	*P*^2 ^= 0.862
	TOTAL	107	63	259	106	

Median household monthly expenditure US$*		36.96	41.3	39.13	39.13	*P*^1 ^= 0.738
						*P*^2 ^= 0.458

N/A = not applicable. *Sample size for mean monthly expenditure estimate: child private = 35, adult private = 21, child government = 87, adult government = 41. *P *values generated by chi-square or Mann-Whitney tests comparing adult^1 ^and child^2 ^participants respectively between clinics.

All 535 individuals enrolled in the study at day 0 had clinically diagnosed malaria but only 396 (74%) had microscopically confirmed parasitaemia. Clinical diagnosis of malaria at both clinics was more reliable for children than adults. 77% of malaria diagnoses in children and 69% in adults were confirmed by microscopy (p = 0.093, χ^2 ^test) (Table 1). Although all enrolled patients were clinically diagnosed with malaria, 41% of children (78/188) and 23% of adults (24/106) were not given prescriptions for anti-malarial drugs at the initial visit (Table 2). Of the 192 patients who were given anti-malarials, 133 (69%) received the nationally recommended AQ/SP combination, 37 (19%) received SP alone and the remainder received monotherapy with chloroquine, quinine or amodiaquine. Retrospective analysis of antimalarial prescriptions for adults with microscopically confirmed malaria, revealed no difference in the anti-malarial drugs prescribed for those with true malaria compared to those with a false positive diagnosis (p = 0.933, χ^2 ^test).

**Table 2:** Malaria treatment prescribed for patients with clinically diagnosed malaria at day 0 (n = 294; treatment regime not known for 18 individuals). SP = sulfadoxine pyrimethamine.

**Treatment Regime**	**Total**	**Adult**		**Child**	
		**Microscopy positive**	**Microscopy negative**	**Microscopy positive**	**Microscopy negative**
**No antimalarial**	**112**	**17 (21.8%)**	**7 (25%)**	**43 (32.3%)**	**35 (63.6%)**
**Antimalarial **	**192**	**61 (78.2%)**	**21 (75%)**	**90 (67.7%)**	**20 (36.4%)**
Amodiaquine/SP	133	44	20	57	12
Chloroquine	12	10	0	3	1
Quinine	1	1	0	0	0
Amodiaquine alone	7	0	0	6	1
SP alone	37	6	1	24	6

**Total**	**294**	**78**	**28**	**133**	**55**

75 (37%) children and 43 (40%) adults still had their original symptoms at day 7 but this had fallen to 33 (16%) and 30 (28%) respectively by day 21. At day 21 but not at day 7, prevalence of original symptoms was significantly higher in adults than in children (day 7 p = 0.933; day 21 p = 0.023, χ^2 ^test). 51 (25%) children and 41 (38%) adults had developed new symptoms such as anaemia, wheezing, cough, convulsions, diarrhoea, vomiting, constipation and earache by day 7 and these persisted to day 21 in 26 (24%) of adults and 15 (7%) of children. The chance of new symptoms occurring was significantly greater in adults compared to children at day 7 (p = 0.024, χ^2 ^test) and day 21 (p < 0.001, χ^2 ^test). There was no significant difference in persistence of original symptoms or in recurrence of new symptoms between patients (either adults or children) with a correct or incorrect diagnosis of malaria at either 7 or 21 days.

**Figure 1:**
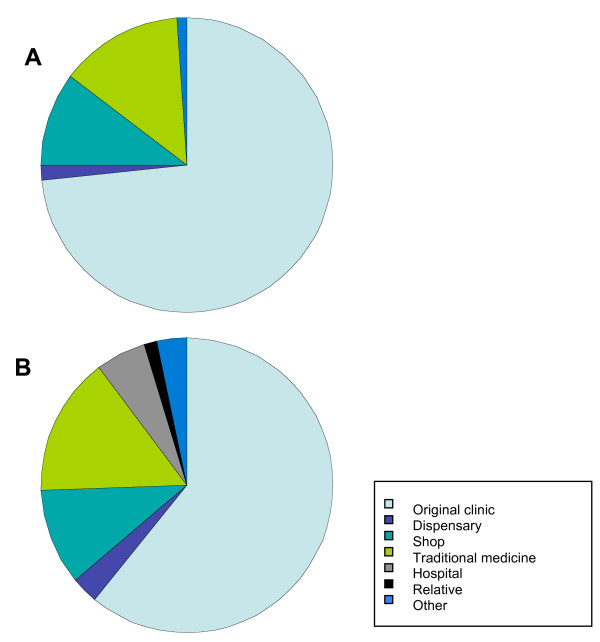
Providers of health care for A. children (N = 108) and B. adults (N = 56) with clinical diagnosis of malaria who sought additional health interactions over the 21-day study period.

The number of times individuals sought any HI during the 21 days after their initial visit varied from 0 (47%; 146/312 individuals) to nine (one individual). For the 53% of patients who had more than one HI, the median number of attendances was two (Table 3). 54% of children had one or more HIs with no difference in frequency between those with or without microscopy confirmed malaria (p = 0.879, maximum likelihood estimation with adjustment for overdispersion). 46% of adults with microscopy confirmed malaria had ≥ 1 HIs compared to 67% who were misdiagnosed with malaria (p = 0.01–0.06, maximum likelihood estimation with adjustment for overdispersion). The number of HIs by participants who had malaria confirmed by microscopy were also analysed according to whether they received anti-malarial treatment at day 0 or not. There was no difference in HIs between the treated and untreated group for children or adults. Although most repeat health interactions occurred at the original health clinic (61% by adults and 73% by children), 26% of interactions by adults and 24% by children took place at local shops or traditional medicine providers (Figure 1).

**Table 3:** Number (%) of health interactions sought by adults and children over the 21-day study period.

	**Adult**		**Child**	
**Repeat Visit Number**	**Malaria microscopy negative**	**Malaria microscopy positive**	**Malaria microscopy negative**	**Malaria microscopy positive**
0	10 (33)	42 (54)	29 (45)	65 (45)
1	8 (27)	12 (15)	13 (22)	27 (19)
2	4 (13)	13 (17)	4 (7)	30 (21)
3	5 (17)	4 (5)	6 (10)	10 (7)
4	1 (3)	6 (7)	4 (7)	9 (6)
5–9	2 (6)	1 (1)	3 (5)	4 (3)

**Total**	**30**	**78**	**59**	**145**

Healthcare costs incurred by patients over the 21 days following their initial presentation were analysed by clinic because of the different consultation fees charged by the private and government clinics ($0.21 and $0.04 respectively). The median and 25–75^th ^percentile results are presented because the results were not normally distributed (Table 4). Median costs were higher at the private clinic ($0.57, $0.43–0.87 for children and $0.74, 0.54–1.03 for adults at the private clinic; $0.28, 0.15–0.52 and $0.48, 0.24–0.85 at the government clinic). The distribution of expenditure between medications and consultations was similar in adults and children with adults spending 77% on medications and 23% on consultations and children spending 76% and 24%. There was no difference in costs between patients correctly or incorrectly diagnosed with malaria. The number of HIs correlated positively (r = 0.439, p < 0.0001) with the costs incurred over the 21 day period with median costs of HIs by adults and children ranging from $0.28 for the initial visit to $0.51 for 1 HIs, $0.52 for 2 HIs, $0.76 for ≥ 3 HIs.

**Table 4:** Costs of HIs incurred by adults and children who were correctly and incorrectly diagnosed with malaria at the private and government clinics.

**Cost US$**	**Private (US$)**				**Government (US$)**			
	**Child**		**Adult**		**Child**		**Adult**	
	**Malaria diagnosis correct**	**Malaria diagnosis incorrect**	**Malaria diagnosis correct**	**Malaria diagnosis incorrect**	**Malaria diagnosis correct**	**Malaria diagnosis incorrect**	**Malaria diagnosis correct**	**Malaria diagnosis incorrect**
**Median**	0.62	0.52	0.74	0.73	0.39	0.29	0.45	0.48
**25th-75th %**	0.44–0.92	0.44–0.59	0.57–1.12	0.46–0.80	0.15–0.48	0.13–0.72	0.22–0.65	0.37–0.91
**n**	48	13	28	7	97	46	50	23

**Figure 2:**
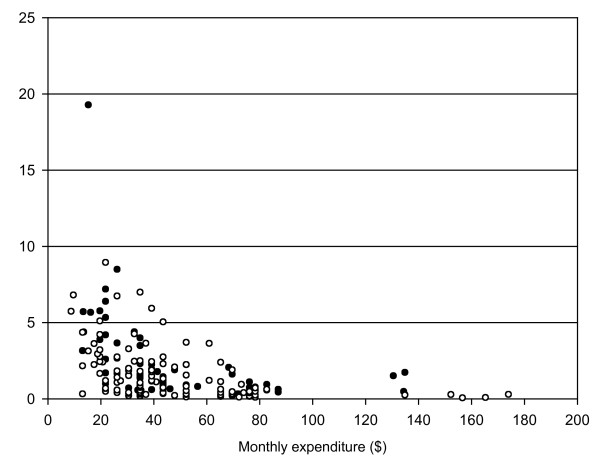
**Adult and child health costs as % of monthly expenditure (US $) (filled circles = adult; unfilled circles = child)**.

Monthly household expenditure varied widely from $9–174 and did not differ significantly between those attending the private and government clinics (p = 0.826, Mann-Whitney U test). Median monthly expenditure was lowest in the households of children presenting to the private clinic (US$36.96) and highest in the households of adults presenting at the private clinics (US$41.3). Health costs over the 21 day study period ranged from $0.1–19.3 (median 1.1) and were proportionally significantly higher (p < 0.001, Mann-Whitney U test) in patients with a household expenditure of ≤ $40/month (i.e. the lowest quintile of household expenditure) compared to those with household expenditure $40–200 (Figure 2). Matching data about loss of earnings (i.e. indirect costs) and monthly expenditure was available for 20 adults and 38 carers. The amount of earnings lost through ill health or by having to take time off work to look after a sick family member varied widely from <$0.01 – $21.7. The median proportion of monthly expenditure that these lost earnings represented was 2.1% for those with expenditures <$40/month and 1.3% for those with expenditure >$40/month (p = 0.229, Mann-Whitney U test).

## Discussion

### Findings

In this study, malaria overdiagnosis rates based on clinical features alone were 23% in children and 31% in adults in rural health clinics in malaria endemic Mozambique. Despite all study patients having a clinical diagnosis of malaria, only 59% of children and 77% of adults were given prescriptions for anti-malarial drugs, and 31% of these prescriptions were for drugs that were not first line treatment in Mozambique's national malaria policy at that time. There is no obvious rationalization for these disparities in prescription rate and drug choice, but they highlight a very worrying issue and it is important to know whether the trend is unique to this setting or representative of a wider scale problem.

A surprisingly high number of patients (53%) sought further health advice after their initial consultation and 25% of these interactions took place with traditional medicine outlets or shops rather than the clinic they had originally attended. Three weeks after the initial consultation, 28% of adults still had their original symptoms and one week after initial consultation 38% of adults had developed new symptoms. These clinical outcomes were significantly worse in adults than in children who overall had a higher proportion of correct malaria diagnoses. Adults who had been overdiagnosed with malaria had significantly more health consultations during the following three weeks than those who had been correctly diagnosed. There was no difference in expenditure on health care during this period between patients correctly or incorrectly diagnosed with malaria and this may have been related to inconsistencies in antimalarial treatment for both groups as well as wide variation in health expenditure between individuals. Total health costs and lost earnings as a proportion of monthly expenditure during the three weeks were significantly higher in the poorest households.

### Comparison with other studies

The rates of malaria overdiagnosis using clinical features alone in this study and the inconsistencies in anti-malarial treatment are very similar to findings from other studies [1,2,10]. This study exemplifies some of the challenges that have been described in the literature in ensuring implementation of new malaria treatment policies. These challenges include encouraging appropriate health-seeking behaviour for diagnosis for fever [11], ensuring drugs reach the poorest households [7] and lack of management capacity to ensure that drugs are only given to confirmed malaria cases [12].

Published studies about the household costs of malaria illness highlight the need to take account of both direct and indirect costs and the disproportionately high economic burden that malaria places on the poorest households [4]. Several studies in developing countries have shown that indirect costs (time missed from work) are around three times higher than direct costs (medications and consultations) [13-15]. All these studies considered the cost of individual episodes of malaria rather than the economic impact on households of repeated health visits due to misdiagnosis. A study of individual malaria episodes from Myanmar, showed that the duration of illness, low income and days lost from work by accompanying persons were associated with higher costs and the authors suggested that the use of confirmatory diagnosis could contribute to minimising the household burden of malaria [14]. The prolonged ill health, and the high frequency and cost of repeated health interactions following clinical diagnosis of malaria found in this study support this suggestion.

## Limitations

Because this was an observational study and confirmation of true malaria infections was done retrospectively, there were no alterations in clinical practice at the health clinics. The inconsistent prescribing of anti-malaria drugs for clinically diagnosed malaria patients, and the occasional use during follow up visits of malaria microscopy results of unknown quality, meant that it was difficult to draw conclusions from data that compared outcomes and costs between those with true and overdiagnosed malaria. Although patients reporting to the formal health clinics in Mocuba were accessed, during the study period, it is recognized that individuals who never access these formal health services may have been excluded and that these individuals are, potentially, most at risk from the consequences of malaria infection.

Health-seeking behaviour was not specifically examined in this study but could confound our results about frequency and cost of health interactions as it is influenced by many factors such as the patient or carers' (usually women) ability to make decisions about types of health care and to access money [16]. Ineffective malarial treatment would have reduced the differential in health consultation frequency between those correctly and incorrectly diagnosed with malaria and despite this the data indicated a strong trend towards increased health consultations in adult patients who were overdiagnosed with malaria.

The true costs of overdiagnosis for malaria negative patients cannot be strictly inferred as there is no way of knowing what would have happened to these individuals if a confirmed negative diagnosis had been available. That is to say, if a slide negative individual had been investigated and treated for a non-malaria cause of their symptoms, what the costs of their diagnosis and subsequent treatment would have been and whether their symptoms would have resolved and how this would have affected the number of repeat visits. It was not possible to answer these questions in our study because it was an observational study so changes to regular practice were not feasible and the health facilities had very limited resources to investigate non-malaria causes of symptoms.

The malaria overdiagnosis rates and costs presented in this study may not be transferable to other contexts where malaria transmission patterns, health-seeking behaviour and health systems differ from those in rural Mozambique. However, the consequences of failing to confirm clinically diagnosed malaria infections, particularly on the poorest households, will be common to many communities in sub-Saharan African countries.

### Implications

Malaria overdiagnosis rates using clinical features alone continue to be unacceptably high in all patients except young children living in areas of intense transmission [17]. Malaria microscopy, the gold standard for malaria diagnosis, has accuracy in routine use of 70–75% and costs less than $0.5 a test but is not usually provided at primary level facilities because it requires trained and supervised microscopists, well-maintained microscopes and systems for checking quality [18]. A simpler diagnostic test, the rapid malaria antigen detection dipstick [17] is becoming affordable (<$1/test), is easy to use and is particularly useful for excluding malaria infection. Even when diagnosis is used correctly, overdiagnosis is likely to occur as it is widely recognised that febrile individuals in endemic areas may have a detectable parasitaemia without suffering from clinical malaria. This study emphasizes that much more needs to be done to evaluate and deploy methods for confirming malaria infection at community level and in encouraging patients to seek accurate malaria diagnosis [11]. Such tools are essential to enable drugs to be targeted to those who truly have disease and their cost-effectiveness increases as anti-malarial drugs become more expensive. Combining accurate diagnosis with efficient strategies to target at risk groups, e.g. intermittent preventive treatment need to be complimented by protocols for the management of malaria-negative fevers. The current regime of over prescribing antimalarial drugs leads to wasted resources, promotion of drug resistance, prolonged ill health, reduced productivity and increased costs for patients and the health service.

## Conclusion

It has been postulated that possible consequences of malaria misdiagnosis include prolonged ill health, increased costs and loss of faith in formal health services but there is very little evidence to support these suggestions [16]. In this study, adults suffered higher rates of malaria overdiagnosis than children and had more prolonged symptoms and a higher incidence of new problems. This suggests that, for many of these adults their underlying condition had not been adequately managed and was deteriorating. Adults with overdiagnosed malaria had increased rates of repeat health interactions. Repeated interactions incurred higher costs, which placed a disproportionate economic burden on the poorest households and many chose, or were forced, to seek help outside the formal sector.

It is those who are unable to access sophisticated diagnostic services for malaria who are hardest hit by the invisible burden of malaria misdiagnosis. Much more needs to be done to improve and promote access to accurate malaria diagnosis for the poorest communities. For them, the consequences of malaria misdiagnosis contribute to a vicious cycle of increasing ill-health and deepening poverty.

## Authors' contributions

IB and GB conceived and designed the study. TM and GB carried out field work with support from LA and ES. JCCH collated and analysed the data and drafted the manuscript with IB. All authors read and approved the final manuscript.
